# Nanoscale friction and wear of a polymer coated with graphene

**DOI:** 10.3762/bjnano.13.4

**Published:** 2022-01-14

**Authors:** Robin Vacher, Astrid S de Wijn

**Affiliations:** 1 Corrosion and tribology, SINTEF, Richard Birkelands vei 2B, 7034 Trondheim, Norway; 2 Institutt for maskinteknikk og produksjon, NTNU, Richard Birkelands vei 2B, 7034 Trondheim, Norway

**Keywords:** friction, graphene, molecular dynamics, polymer

## Abstract

Friction and wear of polymers at the nanoscale is a challenging problem due to the complex viscoelastic properties and structure. Using molecular dynamics simulations, we investigate how a graphene sheet on top of the semicrystalline polymer polyvinyl alcohol affects the friction and wear. Our setup is meant to resemble an AFM experiment with a silicon tip. We have used two different graphene sheets, namely an unstrained, flat sheet, and one that has been crumpled before being deposited on the polymer. The graphene protects the top layer of the polymer from wear and reduces the friction. The unstrained flat graphene is stiffer, and we find that it constrains the polymer chains and reduces the indentation depth.

## Introduction

Graphene is a two dimensional material that has remarkable properties, both electronic [1–2] and mechanical [3–4]. Even before anything was known about graphene, the mechanical properties were already being utilised in engineering applications. Graphite powder, essentially thick flakes of graphene, has been used as a lubricant additive for over a century to reduce wear and friction during sliding [5–7]. Nevertheless, we still do not understand the wide variety of different mechanisms at play in such systems. During the last few decades, with the development of the atomic force microscope [8] and increases in computing power, it has become possible to investigate more deeply and develop an understanding of the mechanisms that play a role in the friction of graphene [5,9–19]. The effect of graphene coatings and their ability to protect against wear depend on the substrate underneath. So far, they have been studied almost exclusively on metals [20–21].

The tribological properties of polymers coated with graphene have barely been studied on the nanoscale due to the added complexity of the polymer, the tribology of which, even without any coatings, is still not well understood [22–23]. In experiments, the tribology of polymer composite materials containing graphene has been studied with the goal of constructing a self-lubricating material [24]. Saravanan et al. [25] have measured the friction and wear of polymer materials such as polyethylene, polycarbonate, polyoxymethylene, polymethyl methacrylate, polyetheretherketone, and polytetrafluoroethylene [26]. Polymer balls have been rubbed on a steel surface covered with layers of graphene oxide and polyethylenimine. The authors showed that a transfer film of graphene on the polymer leads to lower friction. While to our knowledge there have been no numerical studies of friction on graphene-coated polymers, the graphene–polymer interface has been studied. Rissanou et al. [27–28] show that graphene has a strong effect on the structure and dynamics of the polymer chains near the interface.

In this work, we aim to develop our understanding of the frictional behavior of a polymer coated with graphene by using molecular dynamics simulations of a single sliding asperity at the nanoscale. We show that graphene protects the polymer substrate from wear and identify the mechanism of this protection. We show that crumpling of the graphene has an impact on the friction. In the next section we first describe the simulation setup. Then we move on to discussing our simulations of depositing, indenting, and sliding on graphene. In the final section, we draw some conclusions.

## Simulation Setup

We simulate a slab of polyvinyl alcohol (PVA) coated with a single layer of graphene and a counterbody representing an AFM tip consisting of silicon. The simulations were performed using LAMMPS [29]. We use the same simulation setup for the polymer as in our previous work [23]. We summarise this setup below.

### Interaction potentials

**Figure 1:**
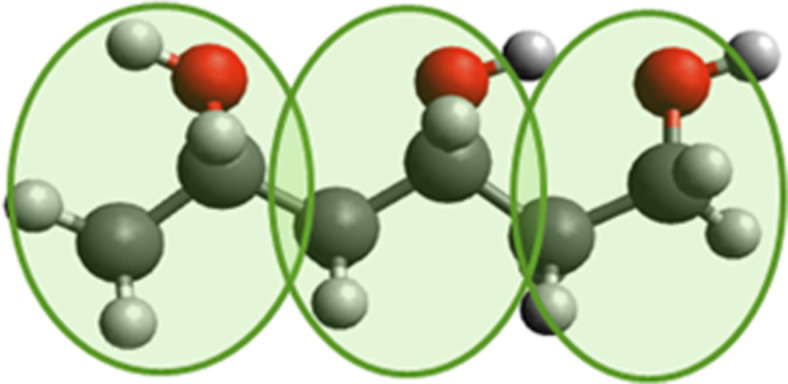
Coarse grained model for polyvinyl alcohol (PVA, C_2_H_4_O)*_x_*). Red atoms are oxygen, dark gray are carbon, and light gray are hydrogen. One green circle represents one coarse-grained particle, which replaces the group of atoms C_2_H_4_O.

PVA is described using a united-atom force field developed by Müller-Plathe and co-workers [30]. Each polymer particle represents a monomer of one structural unit (C_2_H_4_O) (see Figure 1). The nonbonded interaction is given by a Lennard-Jones 9-6 potential,


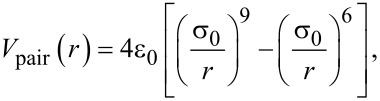


where ε_0_ = 0.0179 eV, σ_0_ = 4.628 Å, and *r* is the distance between the interacting monomers. The bonded interactions are described by a harmonic potential *V*_bond_ = *K*(*r* − *r*_0_)^2^, where *K* = 2.37 eV/Å^2^ is the stiffness and *r*_0_ = 2.6 Å is the equilibrium bond length. The bending potential is approximated by an angular potential described in a table format.

For graphene we use the AIREBO-M potential developed by O’Connor and co-workers [31]. It is an empirical many-body potential that is directly implemented in LAMMPS:


[1] $$V=\sum_{i}\sum_{j\neq i}E_{ij}^{\text{REBO}}+E_{ij}^{\text{LJ}}+\sum_{k\neq i,j}\sum_{l\neq i,j,k}E_{kijl}^{\text{TORSION}}.$$

The interaction between PVA and graphene is modelled using a Lennard-Jones 12-6 potential and the Lorentz–Berlot mixing rule, so that σ_1_ = 4.025 Å, and ε_1_ = 0.015066 eV. We model the interaction between the silicon tip and graphene using a Lennard-Jones 12-6 with the same parameters used by Li and co-workers [32]. ε = 0.092 eV is the depth potential, and σ_2_ = 3 Å is the distance at which the potential is equal to zero. In our system, the tip and polymer are never in direct contact. They are always separated by graphene. We therefore do not need to model their interactions. However, to be sure that no extremely unphysical events can occur, we have used the same potential as for the non-bonded polymer–polymer interaction.

The masses of the particles were chosen to be equal to 12.01 g/mol for the carbon atoms of graphene, 44.17 g/mol for the monomers in PVA and, 2.8 g/mol for the particles of the AFM tip. This leads to a fairly small total tip mass. While this is not entirely physical, such a low mass will help speed up the dynamics and damping of the tip and save computation time without compromising the results [32]. We simulate the system with a time step of 1 fs.

### Substrate cooling and characterization

We start from a box with periodic boundary conditions in the *x*- and *y*-directions (with sizes of 428 Å and 285 Å, respectively), filled up with PVA molecules placed randomly and constrained by hard walls in the *z*-direction. The box contains about 250,000 monomers in chains of 50. Because there are overlaps, we initially give them no interaction. To remove overlapping gently, we first applied a nonphysical soft hybrid interaction potential, for 0.25 ns to remove particle overlapping, and then slowly ramp up the potential over a period of 0.25 ns to the coarse-grained potential described in the previous section. The hybrid interaction potential consists of a 12-6 Lennard-Jones potential for the non-bonded interactions and a spring potential for the bonded interactions.

Once we have reached a melt with the correct interaction, we equilibrate it for 0.25 ns in the NVE ensemble. The temperature of the melt at this point is extremely high. To obtain a realistic semicrystalline substrate structure, we cool down the sample using a Nosé–Hoover thermostat with a linearly decreasing temperature, starting at 5000 K down to 220 K with a cooling rate of 75 K/ns. The damping time of the thermostat is 0.1 ps. After this, the temperature is kept constant at 220 K for 4 ns. At this point, we remove the walls and the *z*-direction as they are no longer needed. The density at this point is 22 monomers/nm^3^. We also switch the thermostat to a Langevin thermostat with a temperature of 300 K and a damping time of 0.1 ps. This thermostat is applied only to the bottom quarter of the PVA molecules, and later to the graphene sheet.

To prevent the polymer slab from moving as a result of the external forces during deposition of the graphene, indentation, and sliding, the centers of mass of the chains in the lower quarter of the substrate are tethered to their original positions using springs with spring constant 1 eV/Å^2^.

### Graphene deposition

**Figure 2:**
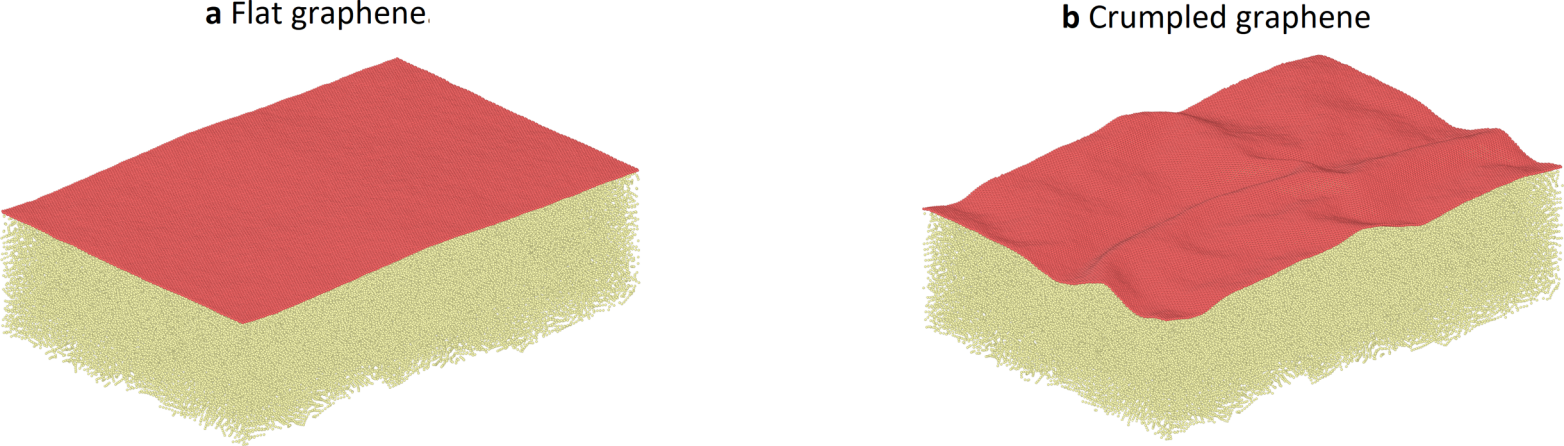
Snapshot of the simulation after the deposition of graphene on the polymer and before indentation and sliding for (a) the flat graphene sheet and (b) the crumpled graphene sheet.

After the solidification of the semicrystalline substrate a layer of graphene is deposited on top. We use two different graphene sheets in our simulations. The first one is a single flat sheet of graphene that has the size of the box (Figure 2a). The second one is also a single sheet, but the graphene has been crumpled through compression along the *x*- and *y*-directions by 10%, which leads to wrinkles on the surface (Figure 2b).

In both cases, we deposited graphene on the surface of the polymer substrate by placing the graphene sheet at around 90 Å from the surface and then applying a force to each of the graphene carbon atoms equal to 0.00005 eV/Å (8.0 × 10^−14^ N) for a period of 75 ps, after which it sits on the surface and has stopped moving. The total normal force applied is around 4 nN (3.3 MPa). Then the force is removed and the graphene sheet stays on the surface due to adhesion.

**Figure 3:**
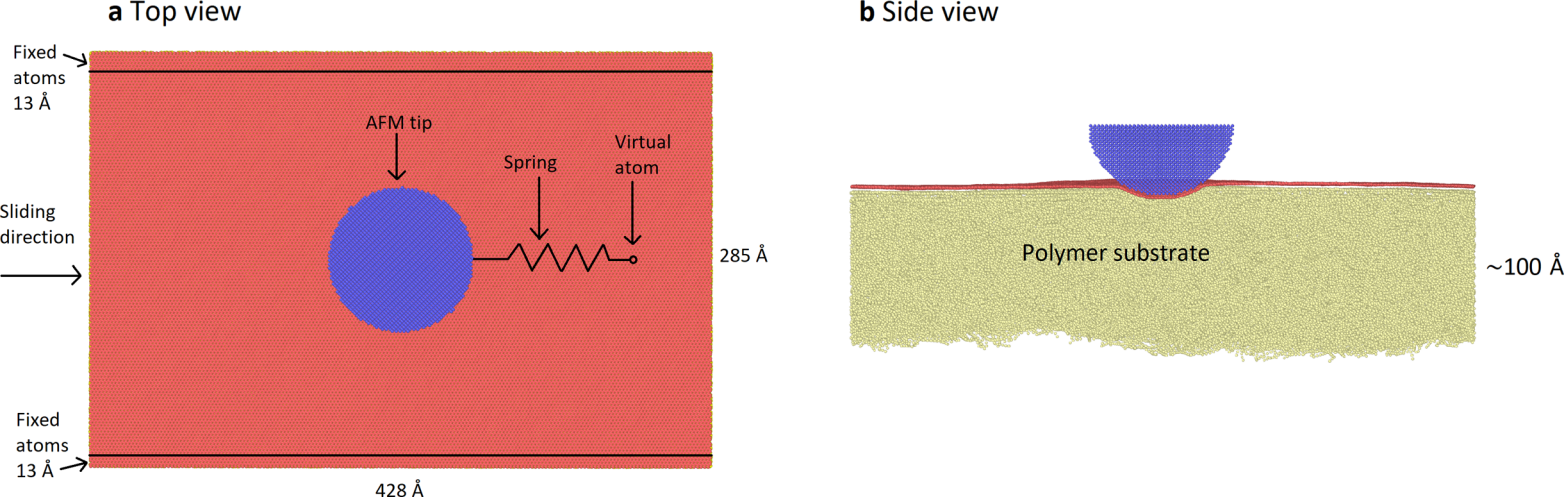
(a) Top view and (b) side view of the simulation. The positions of the fixed graphene carbon atoms are shown. The AFM tip is fixed to a support (virtual atom) via a spring. This support is moving at a constant speed in the sliding direction.

To avoid sliding of the entire graphene sheet over the polymer substrate, we fix the position of some of the graphene carbon atoms during indentation and sliding. The two regions where the graphene carbon atoms are fixed are located in stripes along the *x*-direction, which is the sliding direction, as far away from the trajectory of the tip as possible (Figure 3).

### Indentation and sliding procedure

Once graphene is deposited, we start indenting with a modelled AFM tip. The AFM tip is rigid and consists of atoms arranged in an fcc lattice with a period of 5.43 Å, which is the crystal structure of silicon. A semisphere is cut out from this material. The tip is placed above the surface. A constant normal force is applied to the tip so that it moves towards the surface and indents it. After 1 ns, the tip has reached a stable depth. The tip is then attached to the support with a harmonic spring that acts along the sliding direction. The spring constant is equal to 30 N/m. The support is moving at a constant velocity in the *x*-direction of 2 m/s. We run the sliding simulation for 20 ns, which corresponds to a distance of 100 Å. This takes roughly 6000 CPU core hours.

### Method of analysis

To obtain insight into the collective behaviour of the polymer we investigate averages of, for example, displacement and density. The box is divided into a grid that moves with the tip. During sliding, we bin the individual polymer particles depending on their position in the reference frame of the tip. We then calculate the average of a specific property in each bin.

We also compute the surface roughness. We first divide the box into bins of size σ_0_ in both *x*- and *y*-directions. Each bin is assigned the height of the monomer with the highest *z*-position. We compute the surface roughness as the root mean square height,


[2] $$S_{q}=\sqrt{\frac{1}{A}\sum \sum Z^{2}\left(x,y\right)\Delta x\Delta y}\;,$$

where *A* is the surface area, and *Z* is the height of the particles on the surface.

## Results and Discussion

### Effect of graphene deposition

**Figure 4:**
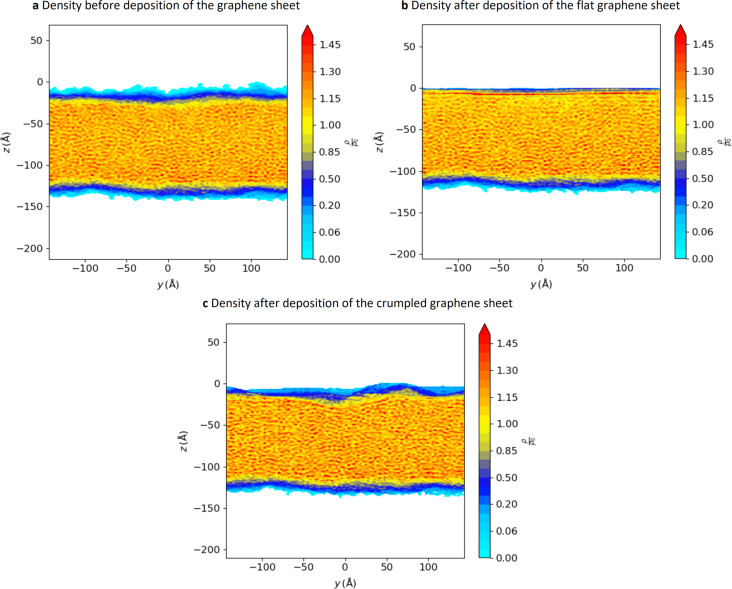
Density of the substrate through the full length of the simulation box (polymer only), (a) before deposition of graphene, (b) after deposition of the flat graphene sheet, and (c) after deposition of the crumpled graphene sheet. The graphene layer affects the roughness and structure of the substrate.

After the deposition of graphene we investigate its effect on the surface. The deposited graphene sheet alters the structure and shape of the surface. This can be seen in Figure 4, where we show the density as a function of the position in a cross section of the substrate for the cases with and without the graphene layer.

We characterise the shape of the polymer surface by the roughness. We computed the roughness of the bare surface, as well as surfaces covered with flat and crumpled graphene sheets just after deposition. Before the deposition of graphene, the roughness of the polymer surface is equal to 0.543 Å. After the deposition of the flat graphene sheet, the roughness decreases to 0.186 Å. After deposition of the crumpled graphene sheet, the roughness changes to 0.581 Å. The flat graphene layer flattens the surface, while the crumpled graphene layer accommodates to it.

In addition to the shape, the structure of the polymer near the surface is affected by the graphene layer. In the case of the flat graphene sheet, the particles of the polymer align in layers parallel to the surface, as can be seen in Figure 4b. In Figure 4b, the red flat region corresponds to a depth at which there is a high density of polymers. A similar effect has been observed in simulations of other polymers [27–28]. For the crumpled graphene sheet, the structure of the polymer is not as strongly affected by the deposition (Figure 4c), although there are some hints of an influence.

### Indentation

**Figure 5:**
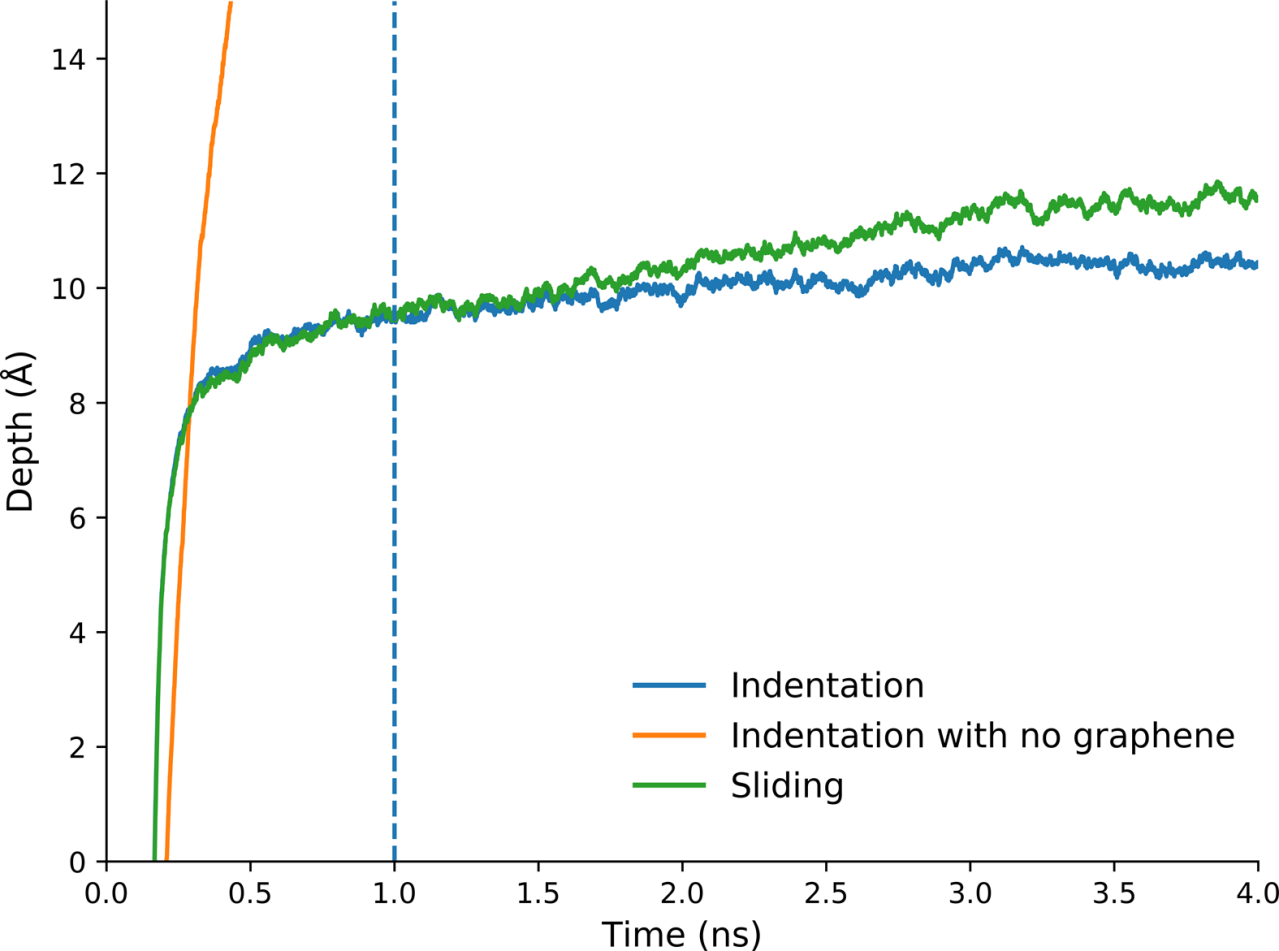
The penetration depth versus time for long indentations, and for the sliding process, for a tip with radius of 50 Å and load of 6.4 nN (4 eV/Å), on the flat graphene. The dashed line represents the time at which we measure the indentation depth and compare this value with other simulations. The sliding process is starting at this point as well. Without sliding the tip does not indent much further, but with sliding it does sink in a little deeper.

After graphene has been deposited, we add the AFM tip to our simulation and indent it into the surface. Figure 5 shows the indentation depth as a function of the time for a normal load of 6.4 nN on the flat graphene sheet. Different loads have been applied in the range of 1–100 nN. The depth was determined as the distance between the lowest atom of the tip and the average height of the graphene sheet before indentation minus the tip–graphene interaction equilibrium distance σ_2_. We have performed this type of analysis for two different tip radii, namely 50 and 100 Å. The sliding starts directly after the indentation process.

We have run a long indentation simulation with a load of 6.4 nN to determine the penetration depth after a long period of time (Figure 5). We observe only a slight increase in depth by around 1 Å between 1 and 4 ns. Thus, we consider the tip to be indented fully after 1 ns. We do note, however, that during sliding the tip sinks into the substrate a bit more than without sliding. We speculate that this may be due to frictional heating, which slightly softens the surface.

**Figure 6:**
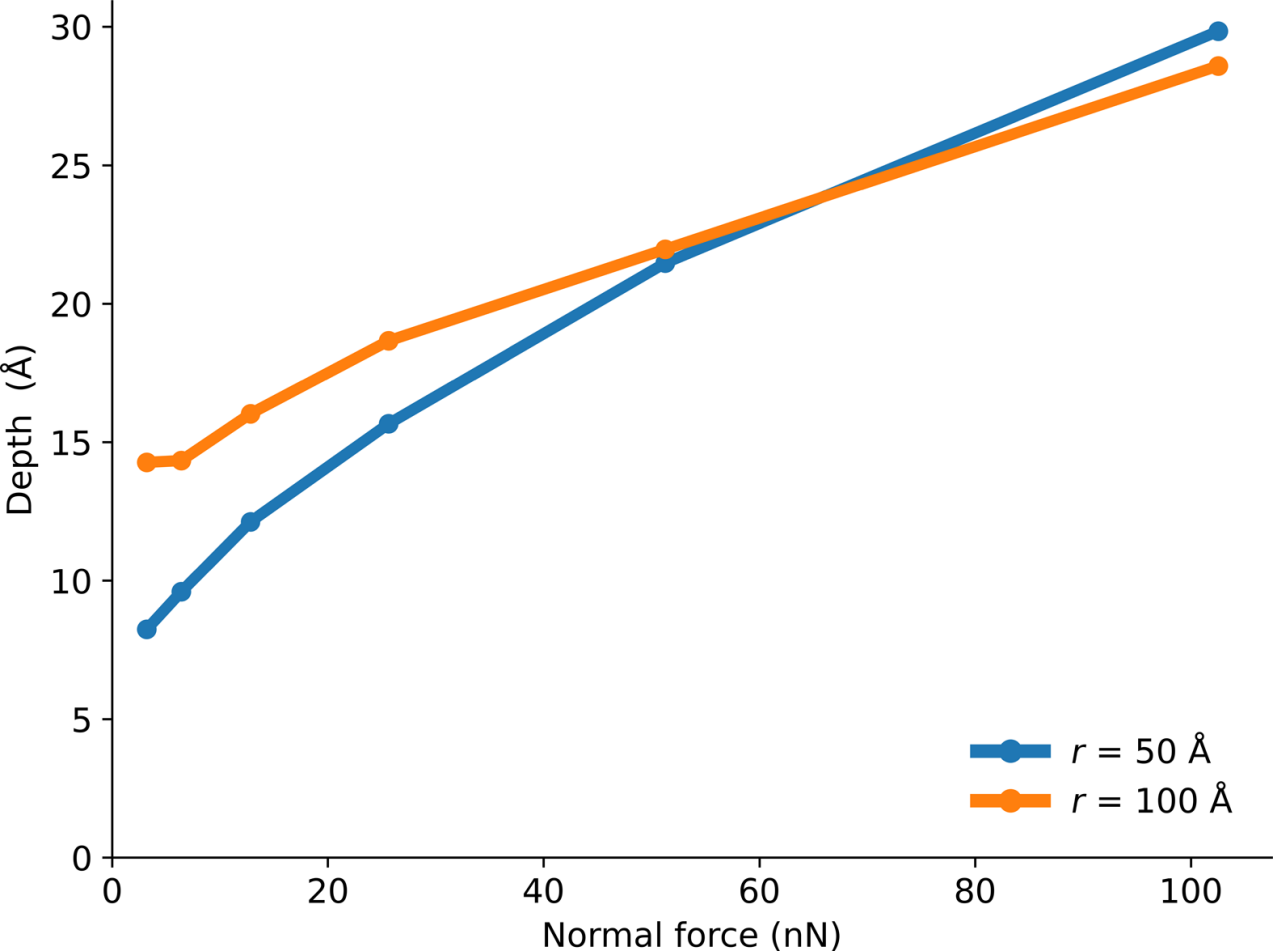
Indentation depth as a function of the normal load for the flat graphene specimen with tip radii *r* = 50 and 100 Å.

**Figure 7:**
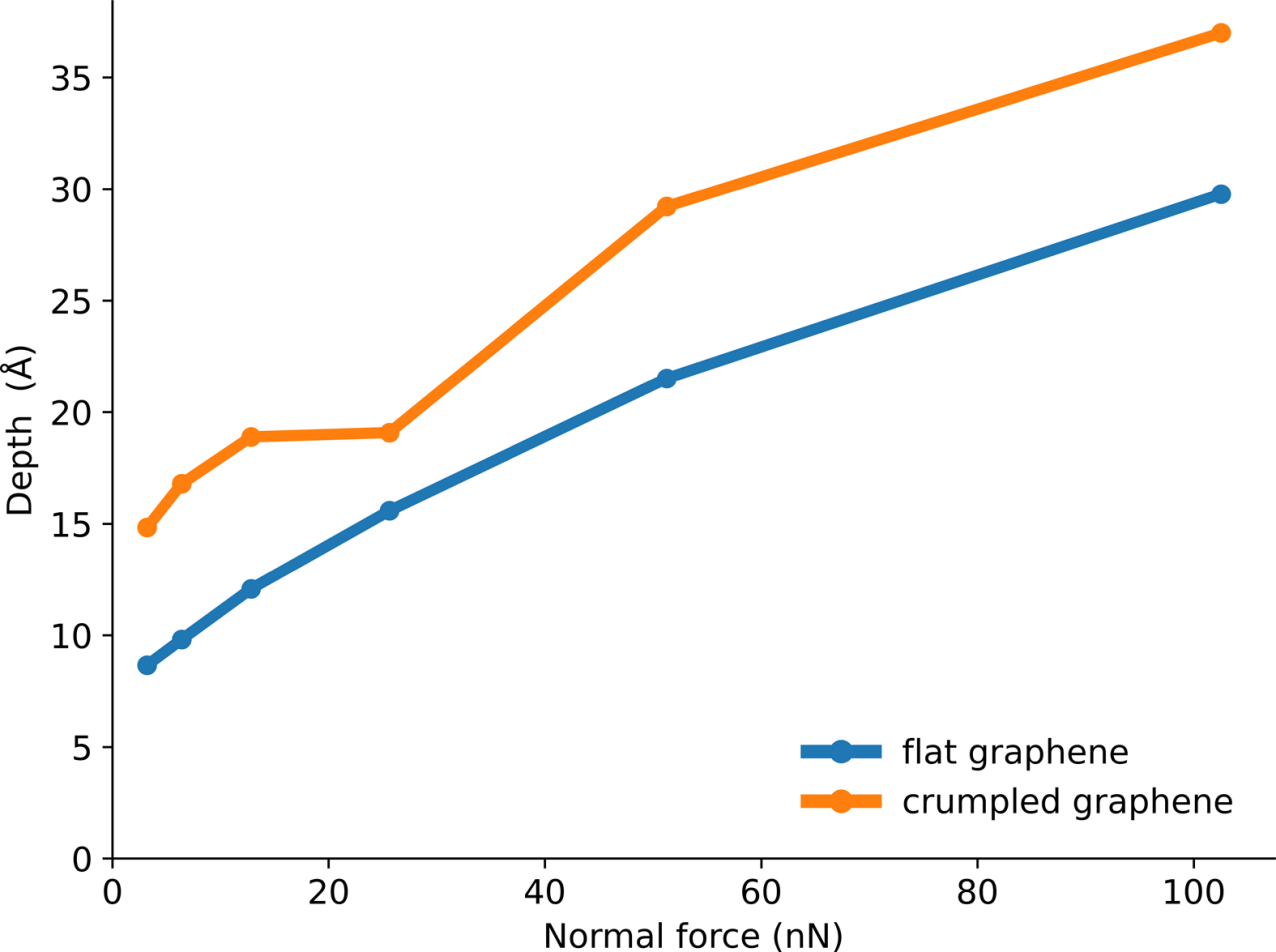
Indentation depth on flat graphene and crumpled graphene layers for different normal loads and a tip radius of *r* = 50 Å.

The indentation depth depends strongly on the load, as expected (Figure 6). At low normal force, the tip with a higher radius penetrates deeper due to adhesion. In this case, the load is small compared to the adhesion force at the edge of the tip. Since a bigger tip has a bigger circumference, it is exposed to a larger adhesion force and a larger total downward force. At higher loads, the smaller tip penetrates further, as it is exposed to larger external pressure. In the case of the crumpled graphene layer, we see a larger indentation depth compared to the flat graphene layer (Figure 7). The tip has more freedom to sink inside the material when the graphene sheet is crumpled (membrane buckling and elasticity) than in the case of flat graphene (stiff membrane).

**Figure 8:**
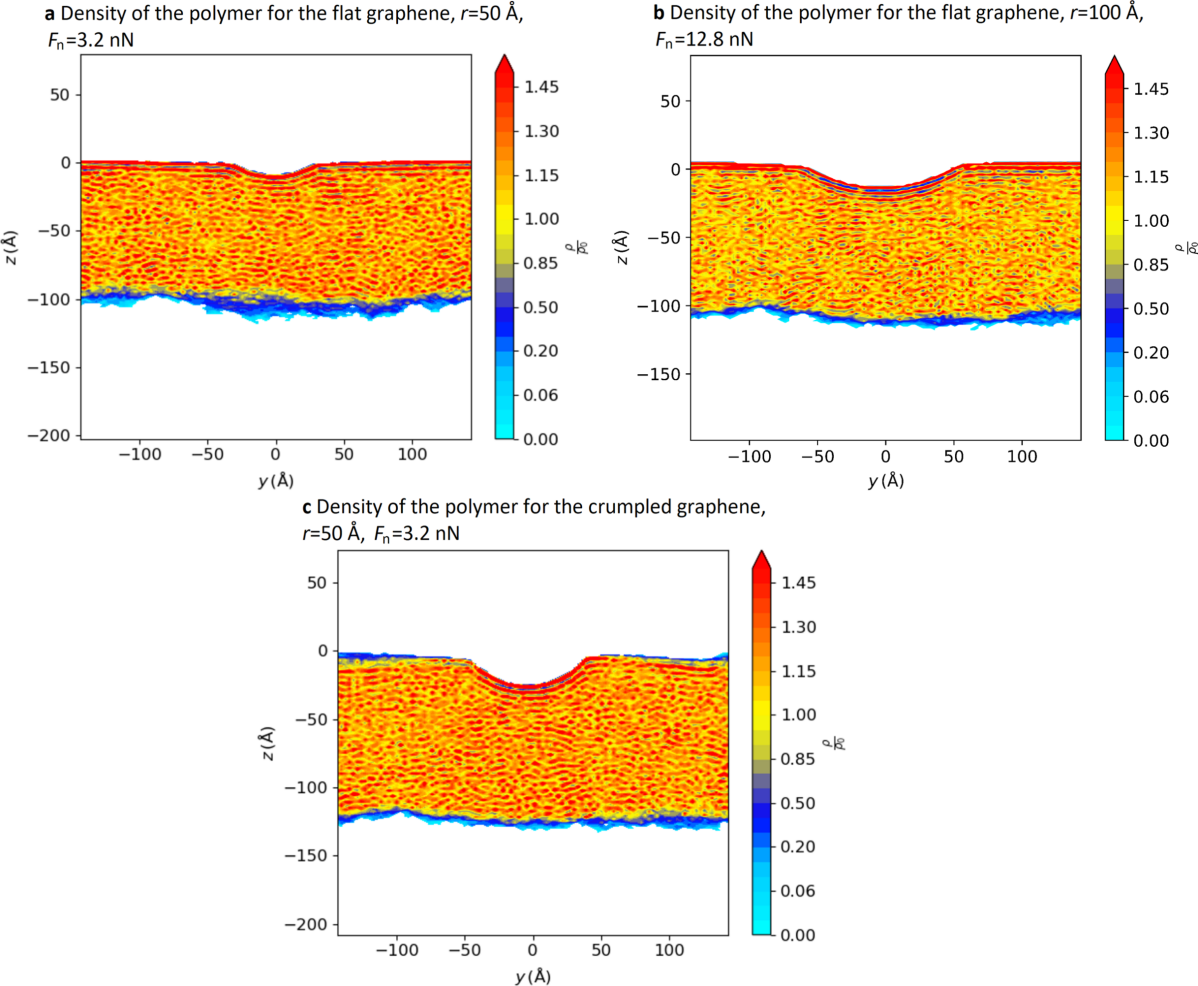
Density maps of the polymer for (a) the flat graphene sheet with *r* = 50 Å and *F*_n_ = 3.2 nN, (b) the flat graphene sheet with *r* = 100 Å and *F*_n_ = 12.8 nN, and (c) the crumpled graphene sheet with *r* = 50 Å and *F*_n_ = 3.2 nN. The cuts are taken right below the middle of the tip on a small thickness (14 Å). The tip indents further on the crumpled graphene sheet.

**Figure 9:**
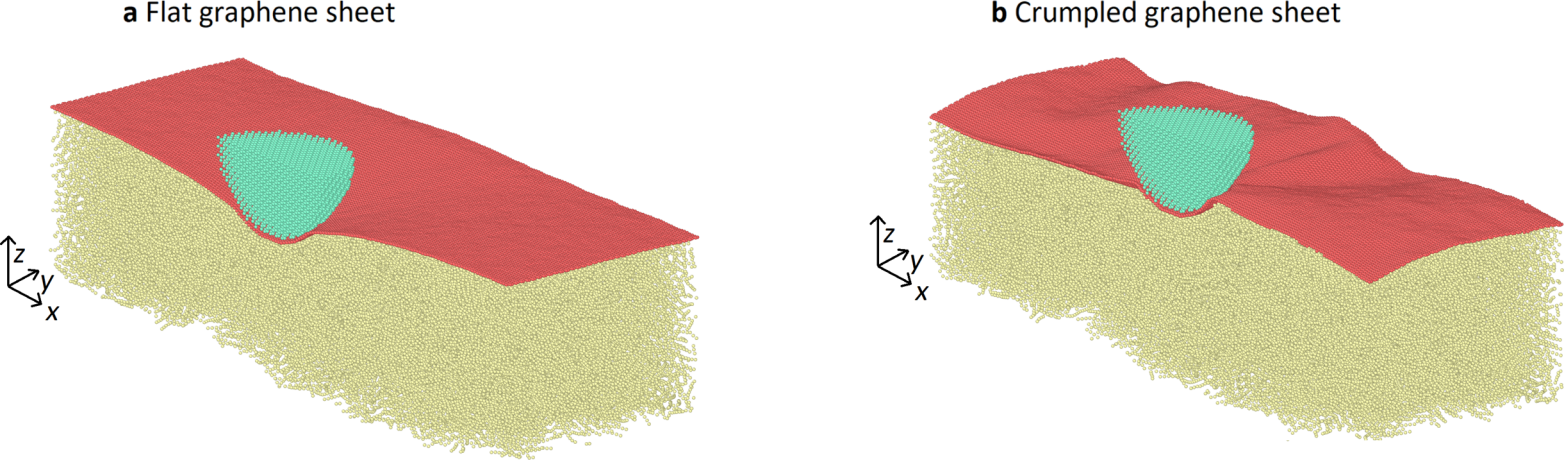
Snapshots of the simulation during sliding for a tip radius of 50 Å and a load of 102 nN (64 eV/Å) for (a) the flat graphene sheet and (b) the crumpled graphene sheet.

Figure 8 shows the cross section of the density under the tip at the end of the indentation process. We can see regular lines of high density right below the graphene layer, which indicate a local reorganisation of the polymer chains. The graphene layer, especially the flat sheet, is also curved away from the tip a little, which plays a role in reducing the local pressure compared to the case with no graphene. This can also be seen in the snapshots shown in Figure 9.

### Frictional forces

**Figure 10:**
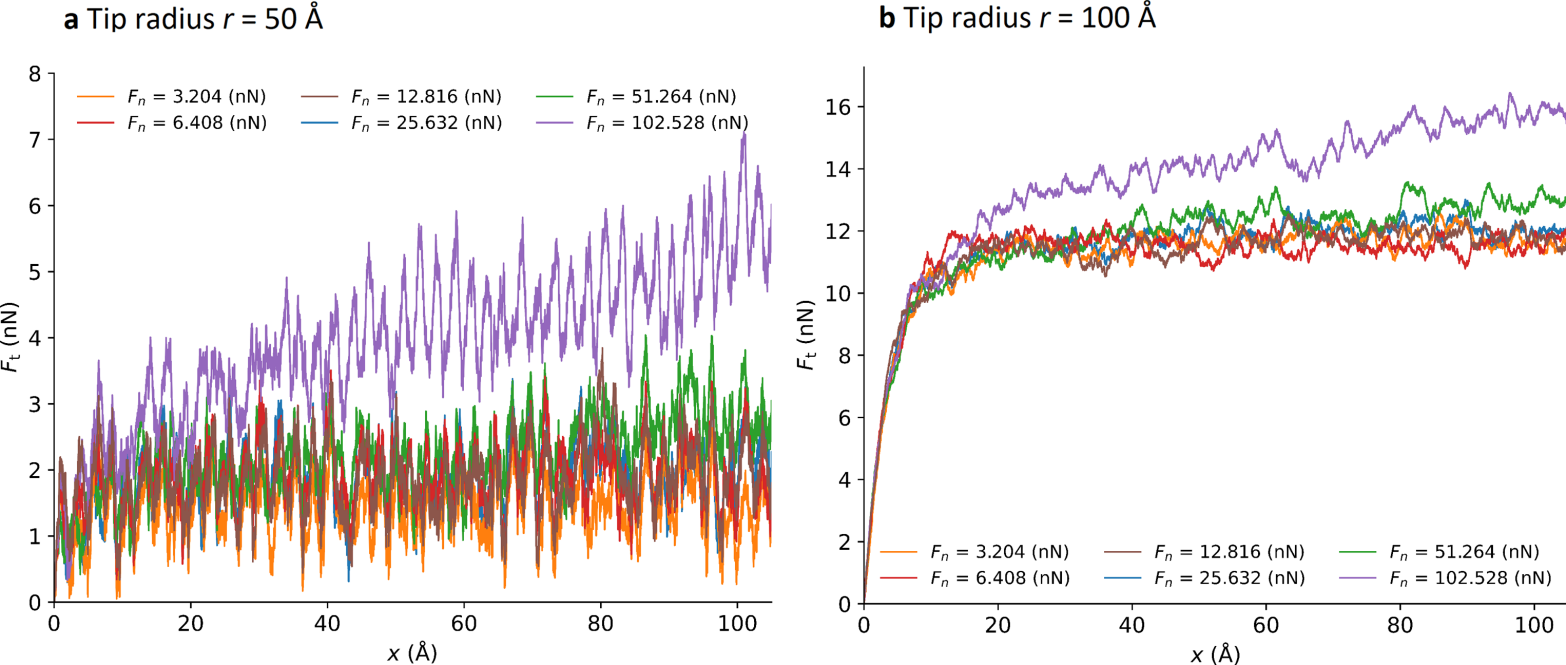
Frictional force as a function of the position of the support on the flat graphene specimen for (a) a tip radius *r* = 50 Å, and (b) a tip radius *r* = 100 Å.

Once the tip is sufficiently indented into the surface (after 1 ns), we start the sliding. Figure 10 shows the lateral force as a function of the displacement of the support in the case of the flat graphene layer.

**Figure 11:**
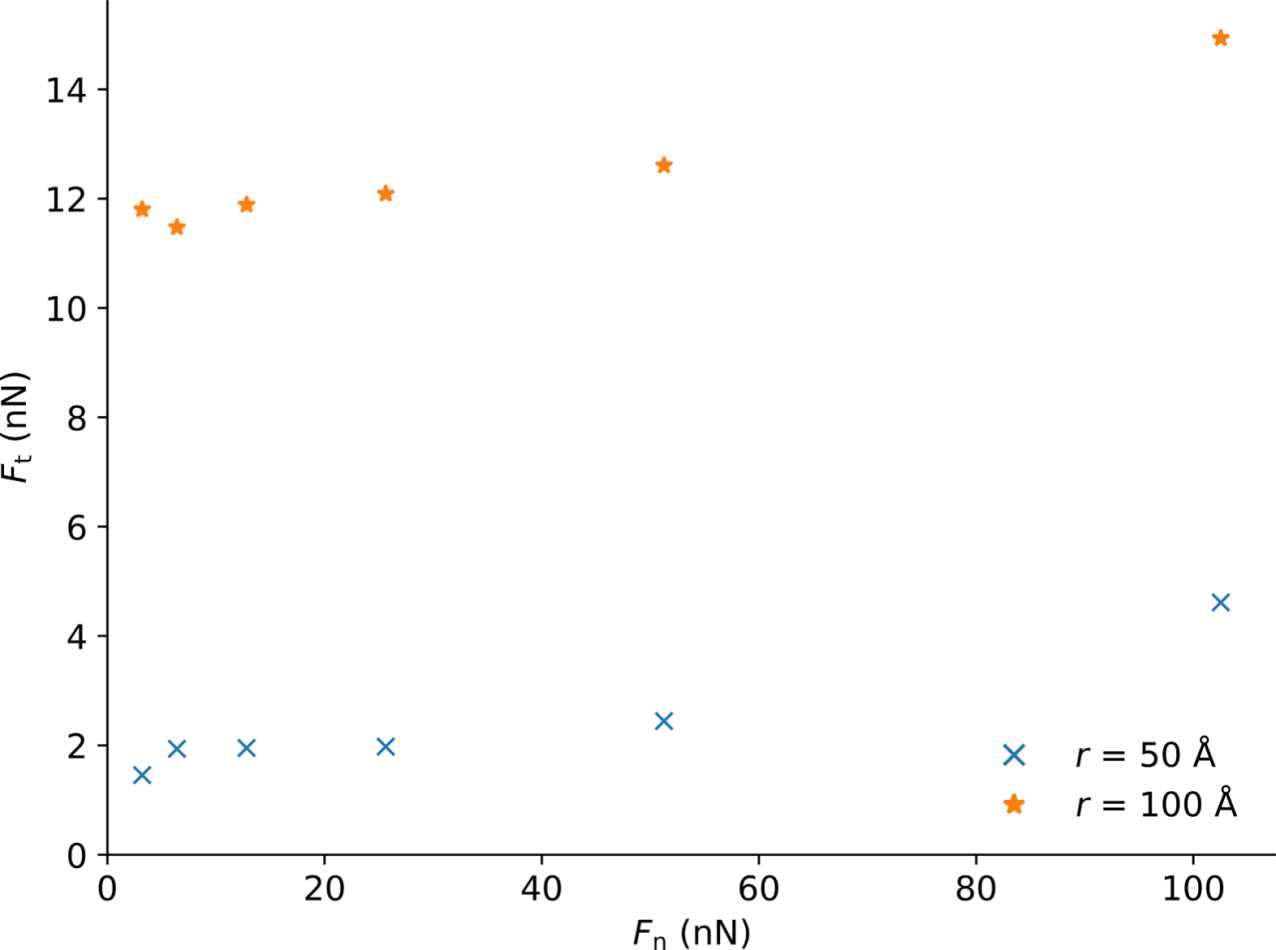
Average frictional force measured between support displacements of 50 and 100 Å as function of the load applied for tip radii of 50 and 100 Å on the flat graphene specimen. For comparison, in a simulation with no graphene, a normal load of 51 nN, and a tip radius of 100 Å, we find an average friction force above 90 nN.

To better highlight the influence of the tip radius, we average the frictional forces between support displacements of 50 and 100 Å. We plot those results as a function of the normal load for two different tip sizes (radius of 50 and 100 Å) in Figure 11. We observe a regular stick–slip motion. The distance between sticks corresponds to one lattice period of graphene. We observe in Figure 10 that for the highest loads the frictional force increases during sliding. This may be due to local frictional heating leading to a change in mechanical properties of the polymer below the tip.

**Figure 12:**
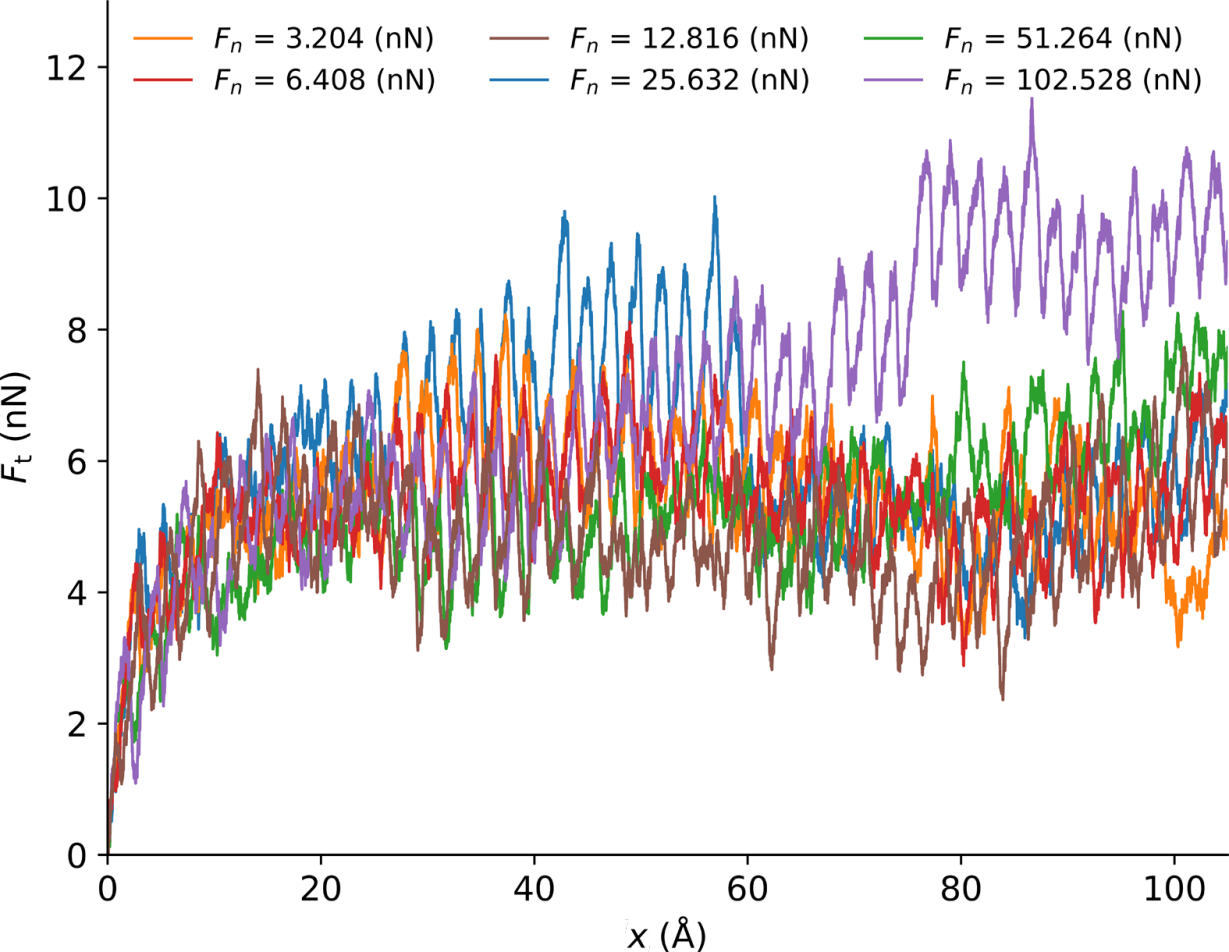
Frictional force versus the position of the support for a tip of radius *r* = 50 Å on the crumpled graphene specimen.

**Figure 13:**
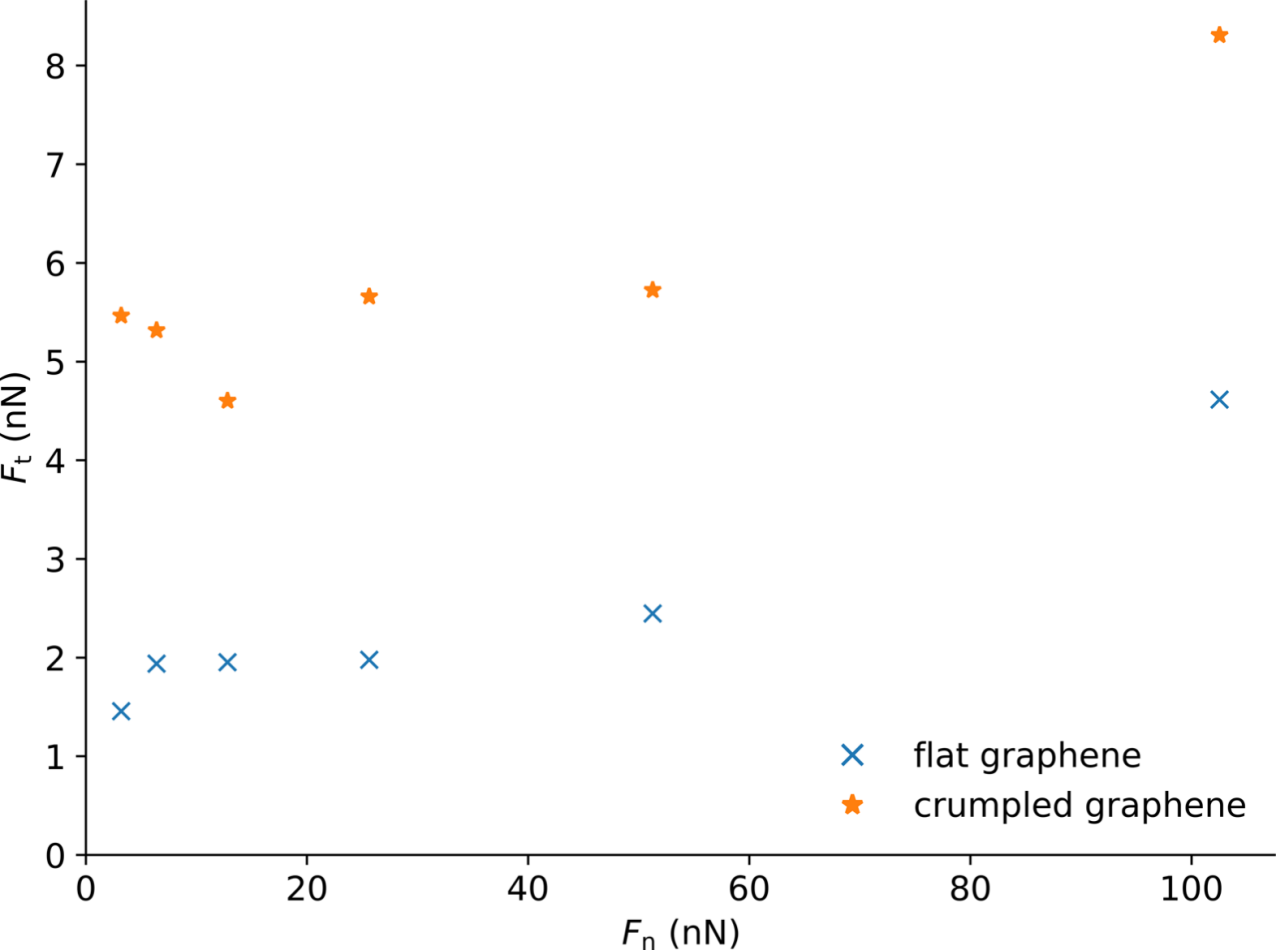
Average frictional force measured between 50 and 100 Å of the support displacement versus load applied for a tip radius *r* = 50 Å on the crumpled and flat graphene.

In the case of the crumpled graphene sheet (Figure 12), the frictional curve is subject to more fluctuations. The calculation of the average frictional force taken between support displacements of 50 and 100 Å (Figure 13) shows the strong impact of the flexibility of the graphene. Again, the higher indentation depth of the tip leads to a stronger frictional force (2 to 3.5 times).

We compare this to sliding without graphene. In a simulation with no graphene, a normal load of 51 nN, and a tip radius of 100 Å, we found that the tip moves deeply inside the substrate and the average friction force is above 90 nN, almost an order of magnitude higher than with graphene. This clearly shows that the addition of the graphene layer drastically reduces the friction.

**Figure 14:**
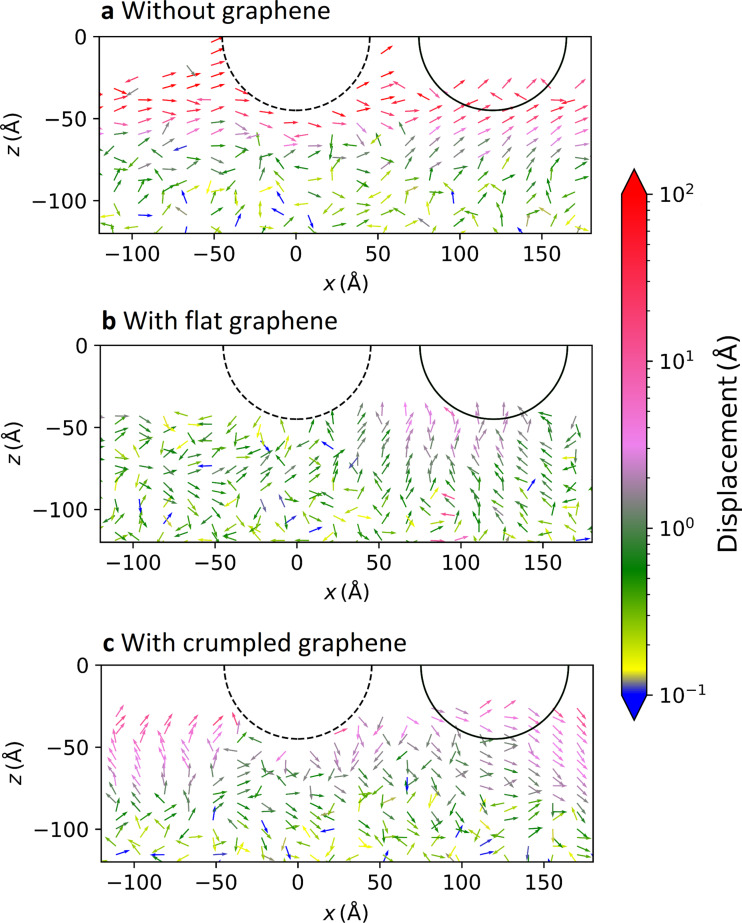
Average displacement of the monomers below the tip during sliding for (a) the case without graphene, (b) the flat graphene, and (c) the crumpled graphene.

To further investigate the mechanisms and observe the effect of sliding on the wear of the polymer material, we compare three simulations, that is, one without graphene, one with flat graphene, and one with crumpled graphene. In this way, we can distinguish between the short-range effects of the protective outer coating, and the more long-range mechanical effects of the graphene layer on the shape of the surface. A normal load of 1 nN and a tip radius of 50 Å were used in all simulations (Figure 14).

To improve the averaging by increasing the total sliding distance, we increase the sliding speed by a factor of 10 to 20 m/s. The displacement vectors are recorded after 0.6 ns, meaning that the support has moved 120 Å. This is indicated by the dashed and solid lines. Without graphene, the vector displacements close to the surface are high and in the sliding direction. This indicates that strong residual deformation remains at the surface because of the shearing of the chains. We observe that the displacements of the polymer are roughly an order of magnitude less when graphene is present. This indicates that graphene efficiently prevents damage of the substrate. The displacements are the smallest in the case of the flat graphene sample, where the graphene sheet is not just protecting the polymer from the tip, but also constraining the chains. Our work thus shows that it is the interaction between graphene and polymer that plays a crucial role in the friction and wear.

The graphene layers we have used, both flat and crumpled, are constrained to remain at a specific length because of the periodic boundary conditions of the simulation box. This means that any elastic stretching of the graphene sheet is limited to a fairly small area. In reality, most of the graphene sheets are larger than the length of our simulation box and depending on how they attach to the surface, they may thus have more length to stretch elastically. Our crumpled graphene sheet, by having a longer equilibrium length than the box, is more representative of completely unconstrained, loose graphene sheets. However, graphene that is bound to the polymer surface, through adhesion or covalent chemical bonds, would behave more like the flat graphene in our simulations and would provide additional protection.

The temperature dependence of friction can potentially allow one to probe different relaxation mechanisms in the polymer [33–34]. In our previous work [23], we extensively investigated the temperature dependence of friction of bare PVA. However, it was not possible to extract information about specific relaxation mechanisms this way because of the dominant involvement of wear. While the graphene coating reduces wear, it does not eliminate it to a degree that we would be able to probe this here.

## Conclusion

We investigate the effect on friction and wear of a graphene coating on a polymer by simulating friction force microscopy experiments with molecular dynamics. A rigid counter-body simulating the tip of the AFM is rubbed against a substrate made of a semicrystalline polymer (PVA) with a graphene sheet on top. Two different graphene sheets have been investigated, that is, a flat graphene sheet that has the same size as the simulation box and a crumpled graphene sheet that has been biaxially compressed by 10%. This allows us to probe some of the mechanisms at play in these systems. Before and after the sheet is deposited on the substrate, we computed the roughness. We observe that the crumpled graphene sheet accommodates to the roughness of the polymer, while the flat graphene sheet reduces the roughness. We also observe a rearrangement of the chain near the surface into a layered structure, indicating that the chains tend to align parallel to the surface. During sliding, the tip sinks slowly into the material. We speculate that this is related to frictional heating in the substrate. For the tip of 100 Å, the sliding delivers about 6 *k*_B_*T*/ps of power. Given the density and the fact that the typical time scale of dissipation is around 1 ps, this is sufficient to increase the temperature of the substrate locally by several kelvin. Near the glass transition, this could lead to significant changes in yield strength. The sinking affects the real surface area and thus also has a noticeable effect on the friction when the normal load is high. The graphene sheets reduce the wear by both reducing indentation and constraining the chains. The displacements of the chains are roughly an order of magnitude smaller when a graphene sheet is present compared to the case with no graphene. The graphene is curved away from the tip; this is especially true for the flat graphene layer. This helps to spread out the pressure and to reduce the local pressure in the polymer. The flat graphene sheet is the most efficient at reducing the friction and wear of the system by this mechanism, as it is harder to penetrate.
